# Implementation Outcomes, Challenges and Prospects of the Capitation Payment Model of Health Financing in Shaggar City, Ethiopia: A Qualitative Case Study

**DOI:** 10.3390/healthcare14213683

**Published:** 2026-10-30

**Authors:** Abera Botore, Mirkuzie Woldie, Kassu Ketema Gurmu, Negalign Berhanu Bayou

**Affiliations:** 1 Department of Health Policy and Management, Faculty of Public Health, Institute of Health, Jimma University, Jimma 378, Ethiopia; mirkuzie.woldie@moh.gov.et (M.W.); kassu.ketema@gmail.com (K.K.G.); negenu@gmail.com (N.B.B.); 2 Shaggar City Administration Health Office, Oromia Regional Health Bureau, Addis Ababa 378, Ethiopia; 3 Fenot Associates, Addis Ababa 378, Ethiopia; 4 World Health Organization Country Office, Addis Ababa 378, Ethiopia; 5 Department of Global Health and Policy, Addis Continental Inistitute of Public Health, Addis Ababa 378, Ethiopia

**Keywords:** healthcare financing, capitation, implementation outcomes, challenges, prospects, Ethiopia

## Abstract

**Background/Objective:** The Ethiopian Health Insurance Service (EHIS) introduced the capitation provider payment model as a pilot in Shaggar City in 2023. To date, no study has been conducted on its implementation status and prospects. Therefore, this study aimed to explore the implementation outcomes, challenges, and prospects of the capitation payment model in Shaggar City, Ethiopia. **Methods:** We conducted a qualitative case study from 1 May to 2 June 2026. Data collection involved 18 key informant interviews, four group interviews with healthcare providers, and three focus group discussions with CBHI beneficiaries. We purposively selected study participants to explore the diversity of views from providers, CBHI implementers, and communities. The data analyses followed hybrid inductive–deductive thematic analysis guided by Proctor’s Implementation Outcomes Framework, supported by ATLAS.ti version 9.1. **Results:** This study included 53 participants. The analysis generated 748 codes, 39 sub-themes, and nine themes: acceptability, appropriateness, adoption, feasibility, fidelity, perceived effectiveness, challenges, facilitators, and sustainability and prospects. Capitation was generally perceived as acceptable and appropriate; however, perceptions of its fairness varied, with providers in lower-category facilities more often describing the payment rates as inadequate or inequitable. Participants reported perceived benefits in financial predictability and beneficiary satisfaction. However, implementation was constrained by challenges including shortages of medicines and supplies, rising service delivery costs, inadequate capitation payment rates, and inaccuracies in household mapping. **Conclusions:** Participants considered the model sustainable and suitable for scale-up only if the identified barriers, particularly inadequate capitation rates and inflation, were addressed. While stakeholders generally perceived capitation as appropriate, acceptable, and feasible, its sustainability and potential for scale-up were viewed as conditional on overcoming these key implementation barriers. Participants also reported key enabling factors, including community ownership, government commitment, evidence-informed implementation, and stakeholder engagement.

## 1. Introduction

Achieving universal health coverage (UHC) is the main health target of the Sustainable Development Goals, which requires health financing to ensure all people access healthcare services without financial hardship [1]. Health-financing mechanisms play a critical role in achieving this goal. Ethiopia is striving to achieve it through implementing various reforms of the health financing system, most notably by the introduction of Community-Based Health Insurance (CBHI) [2,3]. Previously, many countries relied on fee-for-service (FFS) reimbursement, whereby providers were paid according to the services delivered. However, FFS has been criticized for creating incentives for unnecessary service utilization, escalating healthcare expenditures, and increasing administrative burdens [4]. Accordingly, most countries have adopted alternative provider payment models (PPMs), including case-based payment, capitation, diagnosis-related groups, and blended payment models [5].

The Ethiopian government has emphasized the importance of health-financing reforms as a central strategy to improve population health outcomes. Community-Based Health Insurance (CBHI) is one of the mechanisms to ensure people are financially protected from catastrophic health expenditures [6]. The Ethiopian government endorsed and launched the CBHI scheme in 2011, initially piloting it and scaling up to cover 56% of the eligible population. However, its implementation in low- and middle-income countries (LMICs) faces several challenges [7]. Achieving UHC needs not only mobilizing sufficient health financing but also purchasing health services strategically so that available resources generate equitable and quality care. Provider payment mechanisms (PPMs) are a central component of strategic purchasing.

Capitation is one of the PPMs in which healthcare providers get a predetermined payment for each enrolled beneficiary over a specified period, regardless of the number of services utilized. It is expected to enhance effective use of available resources for established service packages of healthcare services while encouraging stronger primary health care (PHC), efficient continuity of care, resource management, and prevention-oriented service delivery [1]. Evidence suggests that capitation can support PHC goals by encouraging cost containment, preventive care, and better management of population health, especially when combined with appropriate service definitions, governance, and models to reduce incentives for under-provision [8].

Capitation has widely been implemented in low- and middle-income countries, especially within PHC reforms and health insurance schemes, to accelerate progress toward UHC by strengthening financial sustainability [9]. Various studies highlight its effectiveness: capitation improves stronger cost containment and financial predictability, improves the availability of essential supplies, reduces unnecessary referrals, and promotes more efficient utilization of healthcare resources [9,10]. However, studies have reported its implementation challenges, including provider dissatisfaction, inadequate capitation payment rates, concerns regarding under-provision of services, reduced service quality, and weak accountability [11,12,13,14,15]. One way of identifying implementation challenges of an intervention is using the principle of implementation science.

Implementation science offers a useful perspective on why interventions fail or succeed in real-world contexts by focusing on how innovations are adopted, accepted, delivered, and sustained in real-world contexts [16]. Proctor et al. distinguish implementation outcomes (acceptability, adoption, appropriateness, feasibility, fidelity, implementation cost, penetration, and sustainability) from service and client outcomes. Implementation outcomes are preconditions for, and intermediate steps toward, the achievement of desired service and client outcomes [17].

Successful provider payment reform depends not only on policy design but also on organizational readiness, stakeholder acceptance, operational feasibility, and the capacity of health systems to operationalize reforms as designed [16]. However, empirical evidence on the implementation process regarding how capitation has been implemented in routine practice remains inadequate in Ethiopia. The available evidence has mainly focused on healthcare utilization, CBHI enrollment, financial protection, and quantitative evaluations of provider payment reforms [18,19]. Currently, Ethiopia has been exploring prospective payment models, particularly the capitation payment model, by initiating a pilot implementation of the capitation payment model in Shaggar City under the CBHI program, with the aim of scaling up and expanding to all regions of Ethiopia based on its feasibility and impact on the health system to fill the gaps of the fee-for-service payment model. Therefore, this study aims to explore the implementation outcomes, effectiveness, challenges, and prospects of the capitation payment model in Shaggar City.

## 2. Methods

### 2.1. Study Design and Setting

This study applied a qualitative case study design in Shaggar City, Oromia, Ethiopia, from 1 May to 2 June 2026. This design supports an in-depth examination of complex implementation processes within a real-world context and helps explore many stakeholder perspectives and beneficiaries on how the capitation payment model is implemented and the challenges faced [20].

Shaggar City is a newly established metropolitan city that encircles Addis Ababa. Administratively, it consists of 12 sub-cities and 36 woredas. Regarding government-owned health facilities, Shaggar City has 35 health centers, one primary hospital, and two general hospitals. Capitation has been implemented in selected health centers of Shaggar City as a pilot starting in July 2023. Under the capitation model, CBHI members are assigned to designated PHCU facilities based on geographical proximity. The facility then receives prospective payments based on facility category and the number of beneficiaries. Facility categorization is based on predefined assessment criteria, including service utilization and comprehensive medical audits [21].

### 2.2. Study Participants and Sampling

The study participants were selected using maximum variation purposive sampling [22] to capture a comprehensive understanding of the implementation process, challenges, and prospects. Participant selection considered various criteria, including positions, years of experience, facility category, level of involvement in CBHI implementation, and other relevant characteristics. Participants were recruited from both health systems across different levels and CBHI beneficiaries. From Shaggar City, participants were drawn from 13 of the 25 health centers involved in the capitation pilot, with representation across facility categories, sub-city levels, and higher-level CBHI stakeholders. A total of 18 key informant interviews (KIIs), four group interviews (GIs), and three focus group discussions (FGDs) were conducted.

The KIIs included health extension workers (HEWs) and health professionals at the health-center level, including primary healthcare unit (PHCU) directors, pharmacy personnel, and finance focal persons. Additional key informants were recruited from sub-city CBHI scheme coordinators and sub-city health office heads. Higher-level stakeholders were also included from the Ethiopian Health Insurance Service (EHIS), EHIS Finfinne Cluster, Shaggar City Health Office and the Oromia Regional Health Bureau. Moreover, selected participants from referral hospitals were included during data collection when emerging findings identified referral pathways and related challenges as important issues requiring further exploration.

Four GIs were conducted with healthcare providers (HCPs) at the health center level, including professionals working in pharmacy, outpatient, PHCU director and laboratory units. One GI was conducted with the PHCU director and the vice director. Even though we had initially planned to carry out the KII with the PHCU director, on arrival, we decided to conduct a GI instead to gain a more comprehensive understanding of the research question, as the director had only recently assumed the position.

Finally, with beneficiaries, a total of three FGDs were conducted, comprising one male group and two female groups, to explore a broad range of perspectives. We selected FGD participants purposively to ensure diversity of perspectives while maintaining sufficient homogeneity within each group to facilitate open and comfortable discussion. Each FGD included 6 to 9 participants who were identified in collaboration with the sub-city health office head, health center directors and health extension workers (HEWs). After recruitment, the participants were again screened based on the predefined inclusion criteria.

GIs and FGDs served different purposes. GIs were held with healthcare workers and were considered practical given healthcare providers’ workload and shift schedules, while allowing each participant to provide a detailed account; the interviewer directed questions to the group and sought each member’s account, with limited interaction among participants. FGDs were held with CBHI scheme beneficiaries; the facilitator encouraged participants to discuss and respond to one another’s views, so that the interaction itself generated data on shared and divergent perspectives [23].

Preliminary review of the data took place during fieldwork. At daily debriefing meetings, the data collectors and the study coordinator reviewed field notes and emerging issues to check data quality, refine probes, assess the depth and breadth of information obtained, and identify areas necessitating more exploration. These daily discussions informed the iterative recruitment of additional respondents. Systematic coding and thematic analysis were conducted after data collection was completed and all recordings had been transcribed and translated.

### 2.3. Data Collection Tools and Procedures

Data were collected using pre-tested discussion and interview guides developed using the relevant literature on provider payment reforms and Proctor’s Implementation Outcomes Framework [8,9,16]. The guides were used to explore participants’ perceptions of the acceptability, appropriateness, adoption, fidelity, feasibility, and perceived effectiveness, as well as facilitators, challenges and prospects of the capitation model.

All interviews and discussions were conducted by four trained data collectors with extensive experience in collecting and transcribing qualitative data. Two experienced supervisors provided oversight of the data collection process. Data collection took place in comfortable places for participants and lasted approximately 40 min to 2:30 h for KIIs and GIs, while FGDs lasted for approximately 1 h and 40 min to 2 h. The interviews and discussions were conducted in the local languages (Amharic and Afaan Oromo) based on participants’ preference, and all data were audio-recorded with respondents’ permission. In addition, field notes were recorded during data collection to capture non-verbal cues.

### 2.4. Data Analysis

The audio recordings were transcribed verbatim and translated into English, and to ensure accuracy, the transcripts were reviewed against the audio recordings before conducting analysis. Data coding and management were done with Atlas.ti Version 9.1.

We followed a hybrid deductive–inductive thematic analysis approach as follows. First, two researchers (A.B. and N.B.B.) independently read the transcripts repeatedly to become familiar with the data and conducted initial open coding. Based on the initial coding, a preliminary codebook was developed and subsequently used to code all transcripts. Additional codes emerged inductively when concepts were identified that the initial codebook did not adequately capture, and these were incorporated into the codebook during coding. The two coders (A.B. and N.B.B.) worked collaboratively to compare coding decisions, discuss and resolve discrepancies, refine code definitions, and merge overlapping codes.

Following coding, related codes were grouped into sub-themes, and conceptually related sub-themes were organized into broader themes. The analysis examined similarities, variations, and contrasting perspectives across respondents to develop a comprehensive understanding of the research objectives. Proctor’s Implementation Outcomes Framework was used as a deductive organizing framework for findings related to established implementation outcomes, including acceptability, adoption, appropriateness, feasibility, fidelity, and sustainability. Other themes, including perceived effectiveness, facilitators, challenges, prospects, and recommendations, emerged inductively because they captured important findings not fully represented by the selected Proctor implementation outcome domains.

Finally, the findings were presented as themes and sub-themes and illustrated with representative direct quotations. The coding structure, thematic development, and interpretation were reviewed by three senior experts (M.W., K.K.G., and N.B.B.). The team incorporated their feedback through iterative discussion to refine the codes, sub-themes, themes, and interpretation, reaching consensus among the reviewers and coders.

### 2.5. Trustworthiness

Many strategies were used to ensure the trustworthiness of the findings, starting from study conception and addressing the four principles of qualitative study, including confirmability, dependability, credibility, and transferability. First, the data were collected by well-experienced data collectors with well-prepared guides with probing. During data collection, the data collectors summarized the key issues raised and invited participants to clarify, expand and confirm, ensuring the data were accurate and comprehensive. Prolonged engagement was ensured, allowing rapport building with participants and understanding contexts before starting actual data collection. An audit trail was maintained to document all stages of the research process, including participant recruitment, data collection, coding process, and theme development. The coding process, theme structure, and interpretation of the research findings were reviewed and approved by three senior research advisors (N.B.B., M.W. and K.K.G.) to ensure analytical rigor. A detailed description of the study setting and the whole process was provided to help readers understand the applicability of findings to similar contexts. The data were collected from several purposefully selected participants at the community level and in the health system at various positions to provide a comprehensive exploration of the research question. Reporting of the study findings was based on the Consolidated Criteria for Reporting Qualitative Research to ensure transparent and comprehensive reporting of the research process [24].

### 2.6. Positionality and Reflexivity

The research team comprised senior public health researchers with expertise in qualitative research and healthcare financing. None of the research team members are affiliated with the health system in the study settings, except the principal investigator (A.B.). During this study, A.B. headed the Shaggar City Administration Health Office, which is accountable to the Oromia Regional Health Bureau, giving him supervisory oversight of the study participants from the city health system. A.B. was involved from this study’s conception and coordinated the overall study and daily debriefing meetings but did not conduct the interviews or group discussions; experienced qualitative data collectors recruited from outside the study setting collected the data. During data collection, participants were informed that this study aimed to understand their experiences and perspectives rather than assess facility or individual performance.

During analysis, the data collectors who conducted transcription and translation de-identified transcripts, and two researchers [A.B. and N.B.B.] coded them independently; the team also deliberately examined divergent and critical views. His familiarity with the local health system, nonetheless, helped the team to interpret participants’ accounts in context. We used open-ended, non-judgmental questioning to reduce social desirability and potential pressure. The team incorporated reflexivity during analysis through independent coding by two researchers, comparison and discussion of coding decisions, and iterative review of emerging themes against the original transcripts. These collaborative procedures enabled the team to critically reflect on potential researcher influence and strengthen the credibility of the findings.

The other research team members were not affiliated with the health system in the study setting, and none were directly involved in implementing, managing, financing, or routinely supervising the capitation program in the participating facilities.

### 2.7. Theoretical Basis of This Study and Its Operational Definitions

This study was guided by the Proctor Framework [17], which provides a structured approach for evaluating the implementation process of the capitation payment model. This framework informed the research process, including preparation of interview guides, data analysis, and interpretation of results. In this study, implementation outcomes, including adoption, acceptability, appropriateness, feasibility, fidelity, and sustainability, were explored. The dimensions explored and operational definitions for each construct are illustrated in the Appendix A (Table A1). Furthermore, the implementation challenges, facilitators, prospects, and perceived effectiveness were explored.

## 3. Results

### 3.1. Participant Characteristics

This study included 53 participants, with representation across different health facility categories, sub-city levels, and higher-level stakeholders. All invited individuals agreed to participate. The final sample was considered adequate based on the breadth and depth of information obtained in relation to the study objectives, with recurring information emerging across successive interviews and discussions, and additional participants recruited iteratively when further perspectives were needed. These comprised 18 KIIs, four GIs containing 12 participants, and three FGDs. Of the FGDs, one was conducted with men (six participants) and two with women (17 participants). The characteristics of FGD participants are presented in the Appendix A (Table A2).

The KIIs included two HEWs, two finance focal persons, two pharmacy personnel, two PHCU directors, two sub-city CBHI scheme coordinators, two sub-city health office leaders, the Shaggar City CBHI scheme coordinator, the Oromia health bureau CBHI expert, the EHIS Finfinne Cluster CBHI expert, the Ethiopian Health Insurance Service CBHI expert, and two representatives from referral hospitals (Table 1).

In addition, four GIs were conducted with small groups of two to four participants, involving healthcare providers from health centers, including outpatient department (OPD), laboratory, pharmacy, and finance units. One of these GIs was conducted jointly with a PHCU director and vice director at a single health center (Table 2).

### 3.2. Overall Themes and Sub-Themes

The analysis produced 748 codes and 39 sub-themes, which were synthesized into nine main themes: acceptability, appropriateness, adoption, feasibility, fidelity, perceived effectiveness, challenges, facilitators, sustainability and prospects (Table 3).

### 3.3. Acceptability of the Capitation Payment Model

This study revealed that participants perceived the capitation payment model as an acceptable provider payment mechanism, but acceptability varied across specific dimensions. Most participants welcomed the shift from fee-for-service to capitation, and these perceptions are described below as sub-themes.

#### 3.3.1. Provider Satisfaction with Capitation

Participants perceived overall satisfaction with the introduction of capitation, particularly when comparing it with the previous fee-for-service payment model. Health facility managers, providers, and scheme officials consistently described capitation as a more reliable financing approach because advance payments enabled facilities to procure medicines and supplies before service delivery, improving service readiness and reducing operational uncertainty.

*Personally, I am happy with this initiative… I perceive that capitation is valuable in solving the healthcare quality-related challenges that existed during the fee-for-service era.*

(EHIS Finfinne cluster CBHI expert)

Similarly, pharmacy personnel linked satisfaction directly to improvements in medicine availability: “*We receive the money in advance and procure supplies early. Patients can obtain medicines here because stock-outs have decreased. Therefore, I am satisfied with this model*” (KII, pharmacy, Melka Nono).

#### 3.3.2. Perceived Fairness of Capitation

Regarding fairness of facility categorization and payment allocation, participants perceived that facility categorization followed predefined assessment criteria, including service utilization and comprehensive medical audits. For example, a regional stakeholder regarded categorization as evidence-based and transparent: “*We categorized facilities based on service utilization, comprehensive medical audit, and expenditure….*” (Oromia Health Bureau CBHI expert).

However, providers working in lower-category facilities questioned whether the resulting payment differentials fairly reflected actual service demands. They argued that facilities with similar workloads and comparable operating costs often received different capitation payments because of their assigned categories. One participant explained that “*Our health center is Category C while others are Category A, yet we provide similar laboratory services. The costs are almost the same, but the payment differs*” (GI, healthcare provider, PHCU, Sebeta).

Many participants recommended the implication of household mapping for equity, saying that mapping facilitated equitable access by assigning households to nearby health facilities, reducing travel costs and enhancing continuity of care. Mapping was positively perceived in areas where communities were given adequate information and facilities were conveniently located.

A PHCU director explained this as follows: “*When the community receives services from the nearest facility they prefer, they are satisfied…*.” (PHCU Director, Sululta).

*… Communities no longer need to go to general or other hospitals to seek services that should be provided at primary healthcare level. As a result, communities save money of transportation costs, since capitation has created a conducive situation to access primary healthcare services at health centers.*

(KII, Oromia Health Bureau CBHI expert)

However, other participants reported that mapping initially generated resistance, particularly among beneficiaries who preferred hospitals or previously attended facilities outside their assigned catchment areas. Several participants attributed dissatisfaction not to the mapping policy itself but to limited community engagement and inadequate awareness during implementation.

As one CBHI coordinator described, “*Initially the community preferred higher-level facilities. Many questioned why they had to be limited to one health center*” (City Scheme Coordinator).

#### 3.3.3. Perceived Adequacy of the Capitation Payment

Participant perceptions about the adequacy of the capitation payment rates varied by level. Higher levels, such as scheme coordinators and regional participants, perceived the payment rate as adequate, noting it was developed using evidence of service utilization data. They also highlighted that many health facilities successfully operated without financial deficits under the current capitation payment model. “*The payment rate is calculated using several years of facility data…Many health centers have not experienced financial deficits because capitation payments exceeded their service costs*” (EHIS and EHIS Finfinne cluster CBHI expert).

Meanwhile, PCHU-level participants perceived that although the capitation payment had initially been sufficient, escalating service delivery costs had reduced its financial adequacy over time.

One PHCU director explained that “*Previously the payment was adequate, but currently it is not. Medicine prices have increased substantially, and the current capitation rate cannot cover the actual cost of providing services*” (PHCU Director, Burayu).

#### 3.3.4. Predictability of Provider Payments

Regarding perceived predictability, study participants explained capitation payment predictability as a main strength of the capitation payment model. Many participants highlighted that predictability in terms of payment is that payments are made in advance, on time, and regularly according to a biannual schedule, which enables facilities to plan procurement, maintain drug stocks, and organize service delivery without waiting for claim reimbursement.

A sub-city health office leader noted that “*Health centers wanted prepayment because it allows them to purchase supplies before providing services*”(sub-city health office head). Likewise, pharmacy personnel mentioned that “…*I am satisfied with the regularity and timeliness of the payments*” (PHCU pharmacy personnel).

However, some participants reported occasional delays in the second-round payments, indicating that predictability was not always fully achieved in practice.

### 3.4. Appropriateness of the Capitation Payment System

Participants perceived that capitation is an appropriate PPM for PHCU and viewed it as compatible with the objectives of clinical service delivery, CBHI, referral practices, and treatment guidelines. Appropriateness in general was described below as sub-themes.

#### 3.4.1. Perceived Alignment with Primary Healthcare Service Delivery

Participants perceived that capitation was appropriate for supporting routine PHC services. This was due to the fact that advance payments from capitation enabled health facilities to procure essential supplies. They mentioned that the predictable availability of funds prevented financial constraints and reduced stock-outs, which supported improved planning at health facilities and ensured uninterrupted service provision.

*For sure, to provide clinical service capitation is appropriate…the prepayment strategy is the best and most timely approach….*

(GI, HCPs, Mana Abichu)

*…Yes, it perfectly fit, timely payment helped us to continually fill the supply related issues.*

(KII, PHCU, pharmacy personnel, Galan Guda)

#### 3.4.2. Perceived Compatibility with Clinical Guidelines and Benefit Packages

Participants perceived capitation as compatible with existing clinical practice, noting that healthcare providers continued to follow national guidelines including Ethiopian primary healthcare clinical guideline (EPHCG), and established clinical treatment protocols, while delivering clinical services. “*Capitation fits with benefit packages. Capitation encourages health centers to provide services according to established guidelines…*”(Oromia Health Bureau CBHI expert).

Meanwhile, although participants generally perceived capitation as an appropriate provider payment model, some expressed concerns that the current capitation payment rate did not adequately reflect the actual costs of delivering healthcare services.

*…The capitation payment rate does not align with the actual costs of providing services.*

(GI, healthcare providers, Koye Face)

*It functions only as a payment system; it is not comprehensive. It does not have a proper system for service delivery.*

(sub-city-level health office head)

#### 3.4.3. Perceived Compatibility with the Referral System

Participants viewed capitation as compatible with the existing referral system and healthcare hierarchy. According to their perception, capitation supported the established referral pathway and promoted continuity of care within the healthcare system. “*The referral system follows the standard linkage: health centers refer patients to primary hospitals, primary hospitals refer to general hospitals, and general hospitals refer to tertiary hospitals*”(Finfinne cluster CBHI expert).

Some participants also perceived that capitation strengthened collaboration between primary healthcare facilities and referral hospitals by fostering greater coordination and communication in the referral process.

*Under capitation, we [PHCU] have strong collaboration with hospitals such as Beijing Hospital, which provide services for insured patients. These changes came because of capitation.*

(GI_HCPs_PHCU, Koye Face)

### 3.5. Feasibility

Participants perceived the capitation payment model as feasible to implement and viewed it as operationally practical within the existing healthcare system. They attributed this perception to the prospective nature of the payments, which simplified payment processes, supported the continuity of routine healthcare services, and facilitated the advance procurement of essential medicines and supplies. However, they also identified several factors that constrained its implementation. The feasibility theme is further described as three sub-themes below.

#### 3.5.1. Perceived Operational Feasibility of Capitation Implementation

The majority of participants perceived capitation to be operationally feasible and reported that health facilities were able to implement the payment model successfully despite some implementation challenges. According to their perception, advance payments facilitated the timely procurement of essential medicines and supplies, which contributed to the continuity of routine healthcare services.

*Yeah, it is feasible because funds are always raised from capitation in advance; we purchase all necessary materials and fulfill our supplies.*

(KII, pharmacy personnel, PHCU)

Furthermore, facility-level providers perceived that routine clinical services continued uninterrupted under the capitation model. However, they noted that occasional shortages of medicines and diagnostic supplies required temporary adjustments to clinical management practices to ensure the continuity of patient care.

*There are no services that have been interrupted so far. Most of the time we treat patients according to established guidelines, but this cannot be achieved 100%… When first-line drugs are unavailable, we sometimes provide second-line medicines….*

(GI, PHCU, healthcare providers, Sebeta)

However, although facility-level participants perceived the payment model as operationally workable, they emphasized that capitation alone was insufficient to fully finance routine healthcare service delivery. “*If facility revenues relied solely on capitation payments, I do not believe these services could be maintained. However, when other revenue sources accompany capitation payments, services can be sustained*” (GI, HCP, PHCU, Sebeta).

#### 3.5.2. Perceived Organizational Feasibility of Capitation Implementation

Although participants perceived capitation as operationally feasible, they also reported several organizational weaknesses that affected its effective implementation. According to participants, these challenges include unclear program ownership, limited institutional arrangements, shortages of dedicated personnel, and inadequate infrastructure.

*There is scarcity of human resources and problems with structural alignment. Nobody owns the insurance program, so there is no focal person responsible for managing and leading the program.*

(KII, Finfinne-level CBHI expert)

*The structure of claim auditing, evaluation, monitoring, and financial management at scheme level is very low.*

(KII, regional-level expert)

Meanwhile, participants’ perception regarding the human resource feasibility of capitation varied. They reported that insurance-related activities were often added to existing responsibilities without adequate training, staffing, or incentives. Participants perceived shortages of qualified personnel and limited implementation capacity as barriers to effective implementation of the payment model.

*Most of the time it is additional work. We cannot focus on only one mission, so it affects our routine work.*

(KII, health extension worker)

*Since the standards of facilities are not equal, it is difficult to implement.*

(GI, healthcare providers, PHCU, Mana Abichu)

*There is a training and capacity gap, and people have little interest to work in this area.*

(KII, sub-city-level scheme coordinator)

*Capitation does not increase workload because it is simply a payment system and patient flow remains the same.*

(KII, regional-level expert)

#### 3.5.3. Perceived Financial Feasibility

Participants perceived the financial feasibility of capitation as challenging, despite acknowledging that prospective payments facilitated routine operations. They reported that the capitation rate did not adequately reflect the actual costs of healthcare delivery, particularly in the context of inflation and rising prices of medicines, medical supplies, and equipment. According to participants, these financial constraints limited the capacity of facilities to rely solely on capitation financing. Consequently, health facilities were perceived to depend on additional funding sources to sustain routine service delivery. “*If our income depended only on capitation payments, we could not sustain services*” (GI, healthcare provider, PHCU, Sebeta and PHCU-level director).

### 3.6. Adoption

Participants highlighted that capitation was introduced as a government-directed initiative and adopted by most health facilities with interest. While many participants perceived the model positively, some raised concerns, as described in the following two sub-themes.

#### 3.6.1. Willingness to Adopt

Most participants mentioned health facilities as willing and interested in adopting the capitation payment model. One of the health provider participants says that “*we did not accept it simply due to an administrative directive. Instead, we accepted it voluntarily, based on the information we had and our understanding of the model at that time*” (PHCU-level dispensary expert).

However, participants perceived that as the capitation adoption expanded, there was an emerging resistance and non-readiness to uptake the capitation payment model among some health facilities. According to participants, this resistance was driven by concerns about potential implementation challenges, limited preparedness, financial implications, community complaints, and uncertainties regarding outstanding payments.

One PHCU director participant said that “*We had fear and had no readiness. They responded that it is a pilot test and will be reconsidered within six months if it faces challenges…*” (PHCU-level director).

#### 3.6.2. Roll-Out and Preparation

Participants explained a well-structured, multi-level implementation process for capitation that involved activities at the national, regional, city, sub-city, health-facility, and community levels during roll-out and preparation. According to participants, the adoption process included awareness creation through orientation and training, community mobilization, stakeholders’ engagement, membership enrollment, facility readiness assessment and selection, and guideline development. Participants further perceived that the capitation model followed four structured implementation pathways at the time of initiation and introduction: the initial phase was assessment, followed by pilot testing, scaling up, and, finally, universal expansion into other health facilities.

*As a policy, capitation had four phases when it was introduced. The first phase was assessment, followed by pilot. Then it will be scaled up, followed by universal expansion.*

(sub-city-level scheme coordinator)

*The works done during the preparatory phases were vast and included training and discussion with health professionals, discussion with the community, and mapping of the households…the training was given at different levels. …the professionals working at the front were trained…For the community, the awareness was created of which health facility they were assigned to and where to go in the health tier system….*

(sub-city-level health office head)

### 3.7. Fidelity

Participants generally reported that core components of the capitation payment model were implemented as designed. However, they also described implementation deviations and context-specific adaptations, which are described as sub-themes below.

#### 3.7.1. Reported Adherence to Clinical and Service Delivery Standards

Participants consistently reported that clinical care under capitation was delivered according to the EPHCG and the CBHI benefit package, including laboratory investigations, diagnosis, prescribing practices, and a referral process.

One PHCU director mentioned the following: “*We have implemented what is called EPHCG. This standard allows us to serve what the patient needs, including diagnosis, laboratory investigations, referral services, and treatment. The implementation of capitation enabled us to implement this standard*” (PHCU-level director).

Similarly, healthcare providers highlighted that patient management and follow-up practices continued to follow national clinical standards. “*Patient management follows the Ethiopian Primary Health Care Clinical Guideline. All approaches to patient management and follow-up appointments are guided by this standard” *(GI, healthcare providers, Koye Face).

Moreover, participants stated that routine clinical audits, prescription reviews, and supportive supervision were conducted to monitor adherence to treatment guidelines to improve the quality of care. “*We have been auditing whether drugs were prescribed according to the standard and providing feedback at the health center level*” (PHCU-level director).

#### 3.7.2. Reported Adherence to Operational, Financial and Administrative Procedures

The study participants reported high levels of adherence to the operational and administrative requirements, and participants explained that health facilities implemented these requirements in conformity with national healthcare financing regulations.

*Capitation money is directly deposited into the account. The amount is known, monitored, and used only for planned activities according to the guideline.*

(sub-city-level health office head)

Furthermore, the findings revealed household mapping was generally implemented according to established operational procedures through a collaborative process involving HEWs and local administrators. “*Households were mapped by Health Extension Workers together with kebele leaders. Individual households were assigned to the nearest health center based on their preference, with priority given to geographical proximity*” (EHIS and Finfinne cluster CBHI expert).

#### 3.7.3. Reported Deviations from Intended Implementation and Adaptive Strategies

Though participants generally perceived that capitation was implemented according to its intended design, they also identified some deviations. Participants emphasized that these deviations were largely administrative constraints, resource shortages, and limited implementation capacity, but not deliberate non-adherence. For the deviations, they implemented various context-specific adaptive strategies, which are described below (Table 4).

### 3.8. Perceived Effectiveness

Participants described several perceived improvements associated with the implementation of the capitation model, including financial viability, service readiness and equitable access, financial protection, quality of care, community trust and ownership, healthcare-seeking behavior, and beneficiary satisfaction. Details are described below (Table 5).

### 3.9. Implementation Facilitators

According to participants perceptions, four key dimensions are identified as facilitators of the successful implementation of the capitation model. They are discussed below as sub-themes.

#### 3.9.1. Perceived Facility Readiness and Supply Improvement

Regarding facility supply improvements, participants noted that the increased availability of diagnostic tools, procurement capacity, drugs, and community pharmacy integration were identified as enablers by participants. “*This capitation has changed a lot of things in our health facilities. We used this to buy a chemistry machine and a CBC machine. As it is paid once, it is important to implement it in something like the procurement of medicines. It has important roles in solving the problems of the supply of health facilities*” (sub-city-level health office head).

Moreover, participants described the integration of community pharmacies into health facilities as a significant facilitator of the capitation model, ensuring patient satisfaction by providing drugs unavailable at the facility level. This integration preserved the clinical decision-making process. “*When we lack certain drugs, CBHI members can still receive them from our community pharmacy because we have one. Currently, CBHI members are accessing services through the community pharmacy…*” (PHCU-level pharmacy personnel).

#### 3.9.2. Perceived Community Ownership and Sliding-Scale Premium Collection

Participants mentioned community ownership, trust, and sliding-scale premium contribution as key enablers of the capitation model. Participants explained that community trust was facilitated because the CBHI program was deeply integrated into the daily lives of patients, who viewed the CBHI card as their identity card, providing them with peace of mind.

*The community enrolled in it, as it is important to them. I consider it as my own property; If I don’t have insurance, I know I were face problems, fail in pain, depending on others, or die.*

(Furi sub-city women FGD)

*Sliding scale is good opportunity to capitation sustainability.*

(KII, sub-city scheme coordinator)

#### 3.9.3. Perceived Evidence-Informed Implementation and Continuous Learning

Participants agreed that the successful implementation of the capitation model started with evidence. Participants noted that findings and experiences from the pilot provided an important baseline for implementation, facilitating the adoption and operationalization of the model.

*…We started as a pilot, followed and supported it for two years, and used the output of the pilot for scale-up. After monitoring and evaluating this program for two years, we expanded it to other health centers.*

(Oromia Health Bureau CBHI expert)

*Initially, when we began capitation payments, providers received a flat rate…, based on findings from the pilot, we categorized facilities and set payment rates by category….*

(EHIS CBHI expert)

#### 3.9.4. Monitoring and Evaluation

The participants perceived that effective monitoring and evaluation facilitated the successful implementation of the capitation model. Participants described referral audits, feedback collection and analysis, and regular supportive supervision as enablers within the monitoring and evaluation sub-themes.

*… If the health center simply refers to the hospital to use the money paid with capitation, we strictly evaluate this. Everyone is accountable for their actions, as they are evaluated by the health office.*

(hospital-level CBHI focal person)

*We conduct an evaluation of our health center based on the community and the council’s concerns. Then we work on the gaps and make them visit the changes. Based on what they have observed, we also get input.*

(sub-city-level health office head)

*Furthermore, study participants identified multilevel regular supportive supervision as facilitators.*

*Yes, there is a supervision from the sub-city, Shaggar and EHIS Finfinnee cluster. Three of them visited us. The Shaggar health office CBHI scheme team visited us at least three times. We are in the third quarter; they have come twice before, and they will come next. Last year, even they visited us four times. From the sub-city scheme, they were with us monthly.*

(PHCU-level director)

But other participants reported inadequate operational budgets, limited supportive supervision and clinical audits. “*There should be clinical audits and supportive supervision, but we have no budget to conduct them. Transportation is required to follow the program, but there is no financial support for these activities*” (sub-city-level scheme coordinator).

#### 3.9.5. Perceived Government Commitment and Support

This study showed that strong government commitment and financial support were main facilitators of capitation implementation in Shaggar City. Funding from the government to health facilities was viewed as a strong complement to capitation income.

*In Shaggar, we already have a relatively adequate budget allocated from government treasury to health sector which was, much higher than other Oromia zones and Towns. This budget may mask the problem of capitation. If the budget allocated from city administration was not adequate, solely relaying on capitation would risk the closure of health centers, especially the rural. What I want to acknowledge Shaggar is that the allocation of adequate budget for health center.*

(sub-city-level health office head)

Another national-level CBHI expert mentioned that “*Political engagement is also strong for example insurance performance is one of the parameters used to evaluate leadership. Achievements in insurance were considered indicators of leadership capability, making political commitment a great opportunity for sustainability and scale up*”.

### 3.10. Perceived Challenges to Capitation Implementation

This study revealed that the implementation of the capitation payment system was limited by multiple barriers operating across the organizational, health system, providers, and community levels. Nine major sub-themes emerged from the analysis: service delivery and referral challenges; community pharmacy implementation challenges; service inequity and continuity of care; emergency service reimbursement challenges; human resource gaps; financial challenges; community engagement and household mapping challenges; facility categorization challenges; and governance, monitoring, and implementation management challenges, which are described below (Table 6).

### 3.11. Perceived Sustainability and Prospects

#### 3.11.1. Sustainability

Most study participants believed that the capitation payment model has the potential to be sustained over time. Their confidence was primarily based on the perceived profits gained since implementation, including predictable prepayment, improved service delivery, growing CBHI enrolment, and alignment with the objectives of the CBHI program.

As one participant explained, “*I believe capitation will be sustainable. The reason is one: it is achieving the mission and vision of CBHI. We are observing the tangible results…*” (PHCU-level director).

Another participant emphasized the role of community ownership: “*I think it will be sustainable. As the community awareness is increasing, even if you want to stop it, I don’t think you’ll be able to stop it… I think it will be sustainable, based on the benefits…*” (CBHI focal person, hospital).

Participants expressed confidence in the concept of capitation; however, they perceived its long-term sustainability as contingent upon addressing key implementation gaps, predominantly those related to inflation, payment rates, household mapping, facility categorization, and financial viability.

One healthcare provider noted that “*If the capitation payment rate continues as it is, service provision will not be sustainable… Without compensation or revision of service prices, facilities will eventually reach zero or even negative balances, making service provision unsustainable.*” (GI, healthcare providers, Sebeta).

Likewise, a finance manager stated that “*The capitation is still important, but it will not be sustained if it does not increase its payment.*” (KII, finance manager, Kura Jida).

#### 3.11.2. Perceived Potential Scalability of Capitation

Participants generally perceived that the capitation payment model has the potential to be scaled up beyond the pilot implementation sites. Some participants also highlighted that expansion had already begun in Oromia, which they viewed as an indication of institutional preparedness for wider implementation.

One participant stated that “*We adopted the initiative and implemented it… its benefits need to be evaluated and scaled up for the nation… We want others to benefit from capitation as we did*” (PHCU-level director, Sululta).

Similarly, another participant remarked that “*If necessary, modifications are made, I believe in its scalability for the benefits of the community… I believe in its sustainability*” (PHCU-level director, Burayu).

#### 3.11.3. General Recommendations for Improvement

The study participants provided various recommendations for the future sustainability of the CBHI capitation program. These recommendations are described as follows.

##### Revision of the Capitation Payment

Participants perceived the current capitation payment rate to be inadequate relate to the current rising of inflation and the escalating costs of medicines and medical supplies. They emphasized the need for a revised capitation payment rate that is informed by evidence, and incorporated performance-based reimbursement mechanisms, and improved revenue sharing arrangements to ensure the provision of quality services to patients.

One participant from GI stated that “*To achieve this, capitation payments should be revised to reflect current market prices. … capitation payment rates should not be based solely on household contributions but should also take into account the actual market prices of these items”(GI, HCP, PHCU, Sebeta)*. Additionally, a finance expert mentioned that “*…the payment rate for the provider/health center should be improved; at least more than half of what is collected from beneficiaries should be paid to the health center*” (KII, finance, Kura Jida).

##### Reform of Household Premium Contributions and Renewal

The study participants perceived that revising household premium contributions would be necessary to sustain facilities’ resilience and ensure provision of essential health services. Additionally, participants also perceived the existing renewal period as challenging for members, noting that it could contribute to lapses in enrolment and interruption in service utilization. Thus, they recommended extending the renewal period to encourage continuity of coverage and improve member retention.

One participant from GI said that “*To further improve and sustain services, household contributions should be increased. I recommend that member contributions be aligned with current market prices and based on evidence*” (GI, HCP, PHCU, Sebeta).

One participant of the men’s FGD stated that “*Another issue is the renewal period it is too short. Why not extend it? We are human, and we can forget. If a longer renewal period were given, it would be better*” (Men, FGD, Burayu).

##### Household Mapping and Facility Categorization Reconsideration

Participants suggested considering household mapping by considering community mobility, proximity, and preferences. Likewise, the facility categorization should be based on objective evidence and updated in a timely manner.

One participant from GI mentioned that “*It would be better if the mapping process were supported by research and based on community interest, to ensure fairness and sustainability*” (GI, healthcare providers, Sebeta).

Another participant from GI stated that “*Many individuals in our city live in rented houses and do not stay in one location for long. Only a few households own their homes. This mobility should be considered during mapping*” (GI, healthcare providers, Koye Face).

##### Implementation of Risk Adjustment

Participants identified the need to implement risk adjustment within the capitation model implementation. Many participants described the reconsideration of high-risk clients, such as those with chronic illness and local epidemiology of disease. Thus, they suggested the transformation from the current flat-based payment model to a risk-based payment model.

A national-level CBHI expert stated that “*Recommend that instead of payment by facility categorization, the system should shift to risk adjusted payments. Risk adjustment is scientifically proven and considers the sociodemographic characteristics of our communities, making it a sounder and more equitable approach*” (EHIS CBHI expert).

##### Sustainable Innovative Financing Systems and Exempted Service Reconsideration

Study participants highlighted the inadequacy of current household premium contributions and scheme capitation payments in helping health facilities withstand inflation and increasing operational costs. They recommended case-based and hybrid payments for chronic cases to reduce the chronic disease burden, the arrangement of reimbursement payment models for exempted services and community pharmacies, and the development of various income-generating sources for a sustainability program that cannot be achieved through contributions and subsidies alone.

A CBHI expert participant stated that “*There are no financial safeguards in place yet…., insurance sustainability cannot be achieved through contributions and subsidies alone. Therefore, innovative financing model are required*” (EHIS CBHI expert).

One scheme-level participant said that “*…The majority of patients are children under five. Since they are exempted from payment, this affects the health center’s budget. It should be replaced*” (sub-city-level scheme coordinator).

##### System Digitalization

Study participants perceived system digitalization of the program as essential for enhancing the future sustainability of the capitation. They highlighted the importance of integrating digital systems throughout the program implementation process, from enrollment and beneficiary identification to claim management and service provision. According to participants, such digitalization would help to reduce moral hazard, improve operational efficiency, alleviate healthcare providers’ burden, improve transparency, reduce delays in service delivery, and strengthen the financial flow and sustainability of the insurance program.

A cluster-level expert stated that “*If premium collection were automated—for example, through platforms like Tele birr or banking systems—it would be much more efficient and reliable…Expanding the use of digital payment systems like Tele birr and banking platforms would reduce delays, and administrative costs, and ensure timely deposits, ultimately strengthening the insurance program’s financial flow and sustainability*” (EHIS Finfinne cluster CBHI expert).

Furthermore, one participant said that “*…The other it should be digitalized at least with excel; the registration of patient should not be manual*” (sub-city-level scheme coordinator).

##### Community and Stakeholder Engagement

Participants recommended engaging key stakeholders, from high-level leaders, healthcare providers and community leaders, including religious leaders, Abbaa Gadaa, and women’s affairs representatives in household mapping, enrollment, and resource mobilization to ensure the sustainability of the capitation payment model.

A participant from GI stated that “*I recommend involving communities during household mapping to minimize complaints. As a health center, we have no role in mapping—it is done entirely by the sub city. However, cooperation between health centers and sub cities during mapping would help address these issues*” (GI, HCP, PHCU, Koye Face).

Moreover, a regional-level CBHI expert said that “*From stakeholders, their engagement is very valuable to realize financial sustainability engaging in resource mobilization, increasing enrollments to maximize money from premium collection, and finding other funding resources*” (Oromia Health Bureau CBHI expert).

## 4. Discussion

This study sought to explore the implementation outcomes, challenges, and prospects of the capitation payment model, and we found that capitation PPM was broadly perceived as an appropriate, acceptable, adopted, feasible, sustainable and potentially scalable PPM for PHCU. Despite these encouraging findings, the implementation fidelity of capitation PPM was constrained by some challenges. In response, participants described a range of context-specific adaptive strategies to maintain service delivery and identified key enabling factors that supported implementation.

The study findings highlight that capitation was widely perceived as an appropriate PPM for primary healthcare. The study participants described the capitation model as consistent with the objectives of the CBHI program through promoting preventive care, encouraging efficient resource utilization, risk sharing, and supporting equitable access to essential health services [25]. This finding is aligned with the studies conducted in Ghana, in which capitation was perceived as improving workflow optimization, reducing administrative waste, improving efficiency and strengthening primary healthcare by rewarding providers for delivering quality care to enrolled beneficiaries [9].

This study revealed that capitation was broadly acceptable to stakeholders, particularly because of its perceived role in predictable facility financing and its compatibility with PHC objectives. Nonetheless, information from Ghana indicates that the acceptance of capitation by providers varies. Providers expressed different preferences for payment mechanisms, with concerns about how payment arrangements might affect service delivery, shaping their perceptions of capitation [26]. This variation showed that the acceptability of capitation PPM is not intrinsic to the payment model; however, it is influenced by adequacy, fairness, and practical implementation of providers’ perspectives. Therefore, ensuring acceptability needs discussion and engagement of program administrators, providers, and communities, as well as timely solving of issues that arise during the implementation process.

Even though participants described the capitation payment model as conceptually appropriate, its operational feasibility was affected by internal factors, including drug and medical supplies shortages, inadequate human resources, and limited infrastructure. This finding is supported by a global scoping review conducted on capitation implementation, which highlighted that pre-existing administrative structures and primary healthcare structures supported the feasibility of capitation implementation [27,28]. The participants’ perception suggests that the success of capitation implementation reform depends not only on designing advance payments but also on the internal and external factors related to the health system and overall institutional capacity. This is supported by a review conducted on African health-financing reforms, in which provider payment innovations frequently grew out of the development of the health system functions needed to sustain this system [29].

The participants’ perception suggests that the adoption of capitation as a PPM within the CBHI system is important. This is a promising result in implementation, as the adoption signifies the choice to integrate and utilize capitation PPM in standard healthcare practices. However, this does not necessarily mean capitation PPM has become fully implemented and institutionalized consistently across all facilities. Variations in facility capacity, staffing, supplies, and administrative systems influenced how the capitation PPM was put into practice. Evidence also reveals provider payment mechanisms may need additional fine tuning to better fit existing health-system conditions and reduce administrative burdens [30]. Therefore, participants perceived that successful scale-up of the capitation payment model would require ensuring facilities possess the technical, financial and administrative capability to apply the payment model consistently across settings.

Even though respondents generally expressed favorable views of capitation, fidelity was affected by specific deviations from the intended implementation model. Participants reported challenges related to medicine availability, limitations in community pharmacy arrangements, deviations from beneficiary mapping, and reliance on alternative financing sources to address gaps in capitation funding. In contrast, inadequate capitation rates, inflation, rising service delivery costs, and financial pressures associated with exempted services and chronic conditions were primarily described as contextual and financing constraints that affected the feasibility and sustainability of the model, rather than direct indicators of fidelity. These results suggest a possible inconsistency between the capitation model and the realities of the local health-system context. Evidence showed that while capitation can improve control, costs, and efficiency, inadequately adjusted payments might lead to unintended consequences, including avoidance of more expensive patients and under-service [9,31]. Participants’ perceptions showed that ensuring fidelity requires timely adjustments to the payment rate instead of rigidly following a fixed rate. Participants perceived that timely updates of capitation rates based on service-delivery expenses and inflation, along with risk adjustment, would help in matching capitation payments to the genuine financial obligations of providers, and that strengthening community engagement in household mapping could improve the legitimacy, accuracy, and accountability of the payment system. Findings from the LMICs also support that the design of capitation should be sensitive to context and adaptable [32].

This study found that participants perceived capitation as contributing to improving the financial viability of health facilities, providing financial protection for beneficiaries, enhancing service readiness and equitable access, improving the quality of care, strengthening community trust and ownership, and improving beneficiary satisfaction. This finding is consistent with studies conducted in low- and middle-income countries [9,32,33] as well as studies done in Ethiopia [30], in which the capitation model was described as improving budget predictability, highlighting that the capitation payment model is effective in maintaining the financial predictability of the health system; however, it depends on the capacity of the health system to implement it. These findings should be understood as reported experiences rather than objectively demonstrated improvements, and further study is needed to assess the model empirically.

The current study revealed key enablers for the successful implementation of the capitation model, including facility supply improvements, community ownership, evidence-based implementation and government commitment. This perception of participants implies that these facilitators strengthened the facilities to address gaps and deliver high-quality services for the patients. This is similar to a study conducted in Nigeria, which indicated that timely capitation payments were essential for health facilities to maintain stock of drugs [34], and a study conducted in Ghana, which indicated that the capitation model is not aligned with the policy agenda due to lack of higher officials’ commitment [35]. Furthermore, a study conducted on the barriers and facilitators to healthcare provider payment reform also identified stakeholder engagement, policy support, prior experience, and sufficient resource availability as facilitators [36].

Despite its perceived effectiveness, our study identified notable implementation challenges in implementing the capitation model that continue to affect service delivery. This study highlighted interruptions in medicine and diagnostic services; referral management-related problems; difficulties in managing community pharmacies; and financial constraints, such as inadequate capitation payment rates, lack of risk adjustment, mismatch between capitation payments and actual service costs, inflation, the burden of exempted services, and the cost of treating chronic diseases as notable challenges in implementing the capitation payment model. Furthermore, human resource and administrative structure gaps, such as shortages of trained personnel, high workloads, and weak organizational structures, along with inadequate community engagement in household mapping and concerns regarding health facility categorization, were identified as main implementation barriers. These challenges have undermined the intended benefits of the capitation model and have negatively influenced both beneficiaries’ experiences and the overall successful implementation of the payment model.

The findings of this study are aligned with evidence from Nigeria [37], Ghana [35], and Indonesia [38] regarding challenges such as resource constraints, low community and stakeholders’ knowledge and information gaps, fragmented digital systems, and lack of infrastructure, all of which compromise equitable implementation of capitation. This similarity could be due to geographical similarities, as low- and middle-income countries share basic structural vulnerabilities that affect the implementation of capitation.

The study participants identified various recommendations for the sustainability and expansion of the CBHI capitation model beyond this pilot phase. They suggested several measures, including revising the capitation payment rate to account for rising inflation and the escalating costs of supplies; adjusting household premium contributions and renewal periods to sustain facilities’ resilience and ensure provision of essential services; reconsidering household mapping through community engagement and preferences; implementing risk adjustment; implementing innovative financing systems, such as case-based and hybrid payment for chronic cases, additional resource generation and the arrangement of reimbursement modalities for exempted services and community pharmacies; system digitalization; and finally, enhanced engagement with community and key stakeholders. These recommendations serve as significant inputs for the successful sustainability of the model by helping to reduce system misuse, moral hazard, and healthcare providers’ burden, improve transparency, minimize service delays, and strengthen the insurance program’s financial flow and sustainability.

The current findings are comparable to those of studies conducted in Indonesia [38,39] and Ethiopia [40], in which stakeholders’ engagement, specifically healthcare workers, community engagement, and community-based education, was suggested to strengthen the sustainability of the capitation model. Similarly, a study conducted in Rwanda revealed that continuous data monitoring and evaluation facilitated capitation implementation by reducing moral hazards and fraud [41]. Moreover, the current study is supported by studies conducted in Indonesia [42] and Ghana [43], which indicated that the capitation payment rate must be increased and adjusted for cost inflation and risk according to patients’ cases to be sustained. This similarity may be attributed to shared fundamental challenges, such as limited community health literacy and resulting knowledge gaps, which limit active participation in health-related activities across these developing nations.

Participants generally viewed capitation as a promising provider payment model with potential to support the objectives of universal health coverage and the financial sustainability of CBHI schemes. However, they emphasized that broader implementation and scale-up would require paying attention to the operational challenges identified in this study. Participant-informed recommendations included strengthening community engagement during household mapping; periodically reviewing capitation payment rates in light of inflation and changing service delivery costs; strengthening evidence-based facility classification; improving monitoring and evaluation; clarifying and strengthening linkages with community pharmacies; progressively digitalizing enrollment, premium collection, and beneficiary management; and strengthening the capacity of healthcare providers and scheme administrators. These recommendations should be interpreted as implementation considerations arising from participants’ experiences, rather than as demonstrated policy effects.

Evidence shows that there is no single best way to design capitation; rather, payment systems need to be tailored to the specific context of the health system [9]. Accordingly, payment structures should be customized to fit local epidemiology, demographic patterns, and economic circumstances. For Ethiopia, this suggests that future improvements to the capitation model should move beyond a uniform payment method toward a more adaptive reimbursement approach that considers variations in population health needs, service utilization, and rising healthcare costs. Such reforms would promote fairness among providers, reduce financial risks, and ensure the long-term sustainability of the CBHI program.

### Strengths and Limitations of This Study

This study has several strengths. First, it is novel; it is the first qualitative study conducted in the Ethiopian context to explore the implementation status of the capitation model under the CBHI program using the Proctor Implementation Outcomes Framework. Second, this study engaged diverse stakeholders and used multiple qualitative data collection methods (KII, FGD, and GI), considering maximum variation to enhance the trustworthiness of the findings. Lastly, we applied the COREQ qualitative reporting guidelines to strengthen the rigor of this study.

However, this study has some limitations. First, it was conducted in a single city and within a pilot implementation setting, which may limit the transferability of the findings. Second, as a qualitative study relying on participants’ perceptions, the findings are not intended to provide statistical generalization and may have been influenced by recall and social desirability bias. Third, this study captured perceived and reported experiences rather than objectively measured changes in service quality, utilization, costs, financial protection, or health outcomes. Thus, the perceived effectiveness reported by participants should not be interpreted as demonstrated effects of the capitation payment model. Finally, systematic coding and thematic analysis were not done simultaneously with data collection; thus, formal code and thematic saturation were not assessed.

## 5. Conclusions

This study found the capitation payment model was generally perceived by stakeholders as acceptable, appropriate, feasible and adopted within participating facilities, with potential for longer-term sustainability and scale-up. Participants associated the model with more predictable facility financing and improved capacity to plan and provide services; however, these were reported perceptions rather than objectively demonstrated effects. Participants perceived that implementation was constrained by contextual barriers including inadequate capitation payment rates, cost inflation, absence of risk adjustment, household mapping challenges, the burden of exempted services, and the cost of treating chronic diseases to achieve sustained benefit.

Participants also identified government support, community ownership, improved supply availability, evidence-informed implementation, and stakeholder engagement as important facilitators. They viewed sustainability and potential scale-up as conditional on addressing these implementation barriers. Participant-informed recommendations included periodic review of payment rates, strengthening household mapping and beneficiary engagement, considering risk adjustment and complementary financing approaches, improving digital systems, and strengthening coordination and stakeholder engagement. Further mixed-methods and longitudinal research is needed to assess changes in service quality, utilization, costs, financial protection, and health outcomes, and to examine the conditions under which capitation can be sustainably implemented and potentially expanded to other settings in Ethiopia.

## Figures and Tables

**Table 1:** Summary of KII characteristics of participants in Shaggar City, Oromia, Ethiopia, in 2026.

Characteristics of Participants		Number
Sex	Female	8
	Male	10
Current educational attainment of participants	Diploma/Level IV	2
	BSc/BA	8
	MSc	1
	MPH	7
Total work experience (in years)		Ranges from 1 to 23 years
Position of the key informants	Sub-city health office head	2
	PHCU director	2
	Pharmacy personnel at PHCU	2
	Sub-city scheme coordinator	2
	Finance focal at PHCU	2
	City CBHI scheme coordinator	1
	Oromia Health Bureau CBHI expert	1
	EHIS Finfinne cluster CBHI expert	1
	HEW	2
	Ethiopian Health Insurance Service CBHI expert (EHIS)	1
	Hospital-level CBHI coordinator	2
Total	18	

**Table 2:** Summary of characteristics of GI participants in Shaggar City, Ethiopia, in 2026.

S/N	Sub-City	Facility Name and Its Facility Category	ID Given During Discussion	Sex	Unit
1	Mana Abichu	Keso Wasarbi, C	P1	M	OPD
			P2	M	Pharmacy
			P3	M	Laboratory
			P4	M	Finance
2	Sebeta	Gora Harkiso C	P1	M	OPD
			P2	M	Pharmacy
			P3	M	Laboratory
3	Koye Face	Tullu Dimtu, A	P1	M	Pharmacy
			P2	M	Laboratory
			P3	M	OPD and CBHI coordinator
4	Galan	Tulu Guracha, C	P1	M	Director
			P2	M	Vice director

**Table 3:** Themes and sub-themes generated from the qualitative analysis in Shaggar City, Oromia, Ethiopia, in 2026.

Themes	Sub-Themes
Acceptability	Perceived provider satisfaction with capitation
	Perceived predictability of provider payments
	Perceived adequacy of the capitation payment
	Perceived fairness
Adoption	Perceived willingness to adopt
	Perceived roll-out and preparation
Appropriateness	Perceived alignment with primary healthcare service delivery
	Perceived compatibility with clinical guidelines and benefit packages
	Perceived compatibility with the referral system
Feasibility	Perceived operational feasibility
	Perceived organizational feasibility
	Perceived financial feasibility
Fidelity	Perceived reported adherence to clinical and service delivery standards
	Perceived reported adherence to operational, financial and administrative procedures
	Perceived reported deviations from intended implementation and adaptive strategies
Perceived effectiveness	Perceived improvement in financial viability of health facilities and the scheme
	Perceived improvement in financial protection for beneficiaries
	Perceived improvement in service readiness and equitable access
	Perceived improvement in quality of care
	Perceived strengthening of community trust and ownership
	Perceived improvement in healthcare-seeking behavior
	Perceived improvement in beneficiary satisfaction
Facilitators	Perceived facility readiness and supply improvement
	Perceived community ownership and sliding-scale premium collection
	Perceived evidence-informed implementation and continuous learning
	Monitoring and evaluation
	Government commitment and support
Challenges	Perceived service delivery and referral challenges
	Perceived community pharmacy implementation challenges
	Service inequity and continuity of care
	Perceived emergency service reimbursement challenges
	Perceived human resource gaps
	Perceived financial challenges
	Perceived community engagement and household mapping challenges
	Perceived facility categorization challenges
	Perceived governance, monitoring, and implementation management challenges
Perceived sustainability and prospects	Perceived sustainability
	Potential sustainability
	Perceived general recommendations

**Table 4:** Reported deviations from intended capitation implementation and adaptive strategies in Shaggar City, Oromia, Ethiopia, in 2026.

Fidelity Sub-Themes as Reported	Reported Implementation Deviation	Adaptive Strategy	Illustrative Quotes
Medicine and commodity supply	Sometimes essential supplies were unavailable despite the expectation that facilities would provide the full-service package.	Patients were referred to community pharmacies; prioritized procurement using available funds; borrowing medicines from nearby facilities and exchange drugs with other facilities.	“*When medicines are unavailable, patients obtain the appropriate first-line medicines from our community pharmacy*” (PHCU-level pharmacy personnel); “*…if the drug is near expiration, we shift it to another facility*” (PHCU-level director).
Claim reporting and performance monitoring	Routine claim audits and reports were often neglected because facilities received payments prospectively.	Continuous supervision and orientation were used to reinforce capitation principles.	“*Providers who prescribe medicines outside the protocol receive feedback, and clinical audits were conducted quarterly across all service delivery points*” (PHCU-level director).
Clinical practice	Diagnostic investigations were occasionally omitted because of shortages of reagents or equipment.	HCPs relied on clinical judgment and symptomatic treatment.	“*In such scarcity we made clinical decisions without laboratory confirmation*” (GI, providers, Sebeta PHCU).
Emergency care documentation	Emergency services were provided but frequently not recorded or claimed for reimbursement.	Facilities delivered emergency care regardless of reimbursement while absorbing the associated costs.	“*Facilities treat emergency patients, but they do not record the cases or claim the service costs*” (EHIS Finfinne-level CBHI expert).
Community pharmacy implementation	Community pharmacy did not consistently function as intended because of reimbursement issues, and duplication of medicines.	Beneficiaries were redirected between health centers and community pharmacies depending on medicine availability.	“*The Kenema pharmacy refused the prescription from Shaggar City*” (FGD, women, Furi); “*The main challenge was that community pharmacies stock the same medicines as health-center dispensaries*” (KII, regional-level expert).
Beneficiary mapping	Some of the beneficiaries sought care outside their assigned health facilities, contrary to mapping guidelines.	Mapping records were revised after community complaints, and exceptions were made for patients requiring continuity of specialized care (e.g., HIV and NCD follow-up).	“*After mapping was completed, corrections were made whenever beneficiaries raised complaints*” (hospital-level CBHI focal person); “*Households with special considerations, such as patients with HIV or non-communicable diseases, are assigned based on their preferences because continuity of follow-up care is important*” (OHB CBHI expert).
Financial management	Capitation payment rate was insufficient to cover actual service costs in several facilities.	Facilities supplemented capitation with government subsidies, internally generated revenue, routine budgets, or other income sources.	“*…we use healthcare finance funds and internal revenues to cover the gap*” (PHCU-level dispensary); “*Capitation provides opportunities to manage systems and finances, and allows facilities to consciously consider and control everything within their operations*” (Finfinne-level CBHI expert).
Data and information systems	Reporting relied on manual records, with incomplete documentation and poor data quality.	Excel-based reporting and paper records were used while awaiting digital systems.	“*There is no digital system for identifying mapped households*” (sub-city-level scheme coordinator).

Note: Italic = participants’ quote.

**Table 5:** Reported perceived effectiveness and related service and client outcomes of the capitation payment model in Shaggar City, Oromia, Ethiopia, in 2026.

Sub-Themes as Reported	Summary of Reported Findings	Representative Quotes
Perceived improvement in financial viability of the scheme and health facilities	Participants reported that advance payments improved financial viability of scheme level and facility by ensuring predictable cash flow, allowing facilities to procure medicines and supplies before service delivery, strengthen financial management, and increase internally generated revenue.	“*The capitation initiative is very fruitful for our facility. Since payment was made in advance, we purchased the necessary supplies and serve our community*” (PHCU-level pharmacy personnel). “*Capitation is good for scheme to run and sustain*” (sub-city-level scheme coordinator).
Perceived improvement in financial protection for beneficiaries	Participants reported that beneficiaries experienced reduced out-of-pocket expenditure and catastrophic health spending because essential services and medicines became more accessible through insurance.	“*Insurance has greatly reduced healthcare costs for us. It saved our money and also protected poor households*” (FGD, men, Burayu).
Perceived improvement in service readiness and equitable access	Capitation enabled health facilities to procure necessary supplies in advance, resulting in improved facility preparedness and continuity of service delivery. It also improved equitable access by enabling vulnerable groups and economically disadvantaged households to receive services based on need rather than ability to pay while reducing travel costs through mapped facilities.	*After capitation all these services became available, solving many community problems. Currently, we provided services comparable to hospitals*” (PHCU-level director). “*Capitation has allowed the poorest to receive services equivalent to those received by wealthier people*” (GI, healthcare providers).
Perceived improvement in quality of care	Better availability of necessary supplies enabled providers to follow clinical guidelines more consistently and delivered more comprehensive care. Nevertheless, some participants expressed concern that providers might reduce service provision to preserve prepaid funds.	“*Capitation enabled healthcare providers to provide services according to the standard*” (PHCU-level director).
Perceived strengthened community trust and ownership	Participants reported that household mapping increased community ownership of nearby health facilities and strengthened confidence in both CBHI and primary healthcare services.	“*Community ownership increased because households were mapped under this health center, created the feeling that’ this health facility is mine*” (PHCU-level director).
Perceived improvement in healthcare-seeking behavior	Improved financial protection and service availability encouraged earlier care seeking and increased utilization of primary healthcare services.	“*Previously people sought care only when seriously ill. Now, once they become CBHI members, they seek care even for simple illnesses*” (KII, health extension worker).
Perceived improvement in beneficiary satisfaction	Participants generally perceived that improvements in medicine availability, access, quality of care, respectful treatment and reduced financial burden resulted in higher beneficiary satisfaction.	“*The community is satisfied because there are very few complaints after capitation was introduced*” (KII, health extension worker). “*All clients, including chronic disease patients, were satisfied*” (KII, pharmacy personnel, Galan Guda).

Note: italic = participants’ quote.

**Table 6:** Summary of implementation challenges to the capitation payment system in Shaggar City, Oromia, Ethiopia, in 2026.

Sub-Themes	Description of the Finding	Representative Quotes
Service delivery and referral challenges	Participants stated shortages of medicines and diagnostic services. Referral challenges include delayed reimbursements, and coordination between health center and hospital disrupted continuity of care and reduced beneficiary satisfaction.	*Once clients are referred, they cannot return to obtain medicines from the community pharmacy. This has become a major community complaint” (sub-city-level health office head).* “*Claim requests are submitted for referral services, but payment is still not made” *(sub-city-level scheme coordinator). “*There is no ultrasound service. The main problem is drug unavailability*” (FGD, women, Koye Face sub-city).
Community pharmacy implementation challenges	It is poorly integrated into the capitation system due to unclear contractual arrangements, inconsistent reimbursement model, duplication of services, restrictive beneficiary mapping, and continued medicine shortages.	“*Community pharmacies were supposed to provide medicines unavailable at health centers, but now they stock the same medicines. Instead of complementing services, they are creating conflict” *(sub-city health office head).
Service inequity and continuity-of-care challenges	There is inequitable distribution of beneficiaries and financial resources between rural and urban facilities. Household mapping and delays during CBHI membership renewal interrupted continuity of care and sometimes forced beneficiaries to pay out-of-pocket.	“*During the membership renewal period, we have to pay out-of-pocket even when we have the renewal receipt. Services should continue without interruption*” (FGD, women, Koye Face).
Emergency service reimbursement challenges	Facilities frequently provided emergency care for beneficiaries outside their assigned catchment areas but failed to document services or submit reimbursement claims, resulting in financial losses and weak accountability.	“*Emergency cases are treated, but they are not recorded and no claims are submitted*” (EHIS Finfinne cluster CBHI expert).
Human resource gaps	Capitation implementation was constrained by absence of dedicated CBHI focal persons, inadequate infrastructure, excessive workload, insufficient training, shortages of trained personnel, and lack of incentives or performance recognition, reducing coordination and staff motivation.	“*The common challenge encountered in Shaggar city is that they do not assign a dedicated focal person for the insurance program*” (EHIS Finfinne cluster CBHI expert).
Financial challenges	Participants identified inadequate capitation payment rates, absence of risk adjustment, inflation, increasing chronic disease burden, mismatches between reimbursement and actual service costs, and unreimbursed exempted services as the most significant threats to financial sustainability.	“*The payment rate per household has not changed for two years… However, the price of drugs on the market is continuously increasing*” (finance manager, PHCU, Kura Jida).
Community engagement and household mapping	Limited community awareness, inaccurate mapping, inadequate beneficiary involvement in household mapping and population mobility.	“*Most of the time households are mapped to far health centers without discussing with the communities… Community engagement is critical during mapping*” (Oromia Health Bureau CBHI expert).
Facility categorization challenges	Perceived lack of transparency and responsiveness in facility categorization.	“*Our health center is Category C while others are Category A, yet we provide similar laboratory services. The costs are almost the same, but the payment differs*” (GI, healthcare provider, PHCU, Sebeta).
Governance, monitoring and implementation management challenges	There is weak governance characterized by limited stakeholder engagement, top-down implementation, inadequate supportive supervision, delayed feedback, poor monitoring and claim auditing, manual information systems, weak digitalization, and limited accountability.	“*There is no digital system for identifying mapped households. Reports are simply exchanged as Excel files through Telegram, and they are not integrated into DHIS2*” (sub-city CBHI coordinator).

Note: Italic = participants’ quote.

## Data Availability

The data presented in this study are available from the corresponding author upon reasonable request due to the qualitative nature of this study and the need to protect participant confidentiality. Full interview transcripts and potentially identifiable information are not publicly available because the detailed professional and organizational information contained in the data may permit identification of participants or institutions. Relevant supporting materials are provided in this article. Further inquiries regarding the data may be directed to the corresponding author upon reasonable request.

## Abbreviations

The following abbreviations are used in this manuscript:

CBHI	Community-Based Health Insurance
COREQ	Consolidated Criteria for Reporting Qualitative Research
FGD	Focus Group Discussion
GI	Group Interview
HEW	Health Extension Worker
KII	Key Informant Interview
LMICs	Low- and Middle-Income Countries
M&E	Monitoring and Evaluation
OHB	Oromia Health Bureau
PHC	Primary Healthcare
PHCU	Primary Healthcare Unit
PPM	Provider Payment Model
UHC	Universal Health Coverage
WHO	World Health Organization

## Appendix A

**Table A1:** Operational definitions of implementation outcomes with dimensions explored in Shaggar City, Oromia, Ethiopia, in 2026.

Implementation Outcome	Definition in This Study	Dimensions Explored
Acceptability	The extent to which the capitation was agreeable, and acceptable as a provider payment mechanism for financing PHC services.	Perceived fairness of the capitation payment rate, facility categorization, household mapping, and premium contribution; adequacy and predictability of payments.
Adoption	The degree to which stakeholders initiated and routinely used the capitation payment system.	Willingness to uptake, resistance, roll out and preparation.
Appropriateness	Stakeholders’ perceptions of the compatibility, relevance and suitability of the capitation payment system for realizing CBHI objectives and strengthening PHC delivery.	Fit with PHC, benefit packages, referral systems, and clinical practice.
Feasibility	The degree to which the capitation payment system could be implemented within existing administrative, organizational, financial, and health system capacities.	Organizational readiness; infrastructure; operational resources.
Fidelity	The degree to which the capitation payment system was implemented as designed.	Adherence to contractual agreements, financial management procedures, referral protocols, clinical guidelines, deviations and adaptive strategies.
Sustainability and scalability	Perceptions of the long-term continuation and financial viability of the capitation payment system within the CBHI scheme.	Financial sustainability; policy support; long-term continuation; scalability.
Perceived effectiveness	Participants’ reported perceptions and experiences regarding the extent to which capitation was perceived to contribute to improvements in service delivery and client-related outcomes	Perceived improvements in financial viability, financial protection, service readiness and equitable access, quality of care, community trust and ownership, healthcare-seeking behavior, and beneficiary satisfaction.

**Table A2:** Background characteristics of focus group discussion participants in Shaggar City, Oromia, Ethiopia, in 2026.

Background Characteristics of the FGD Participants								
Group	Participants	Number of Participants	Age Range	Education	Health Status	Family Size	CBHI Membership Category	Religion
1	Women FGD	9	30–65	Ranged from no formal education to completion of grade 10	Six participants were healthy, while three had chronic diseases	1–5	3 indigents and 6 payers	6 Orthodox, 2 Muslim and 1 Protestant
2	Women FGD	8	35–55	Ranged from no formal education to completion of grade 10	Four participants were healthy, and four had chronic diseases	1–8	5 indigents and 3 payers	2 Orthodox and 6 Muslim
3	Men FGD	6	43–65	From grade 4 to 12 + 1	One was healthy, and five had chronic diseases	2–9	1 indigent and 5 payers	4 Orthodox, 1 Muslim and 1 Protestant
